# Detection of Convergent Genome-Wide Signals of Adaptation to Tropical Forests in Humans

**DOI:** 10.1371/journal.pone.0121557

**Published:** 2015-04-07

**Authors:** Carlos Eduardo G. Amorim, Josephine T. Daub, Francisco M. Salzano, Matthieu Foll, Laurent Excoffier

**Affiliations:** 1 Computational and Molecular Population Genetics Laboratory, Institute of Ecology and Evolution, Berne, Switzerland; 2 Swiss Institute of Bioinformatics, Lausanne, Switzerland; 3 Departamento de Genética, Instituto de Biociências, Universidade Federal do Rio Grande do Sul, Porto Alegre, Rio Grande do Sul, Brazil; 4 CAPES Foundation, Ministry of Education of Brazil, Brasília, Distrito Federal, Brazil; 5 School of Life Science, École Polytechnique Fédérale de Lausanne, Lausanne, Switzerland; 6 Genetic Cancer Susceptibility Group, International Agency for Research on Cancer, Lyon, France; Universitat Pompeu Fabra, SPAIN

## Abstract

Tropical forests are believed to be very harsh environments for human life. It is unclear whether human beings would have ever subsisted in those environments without external resources. It is therefore possible that humans have developed recent biological adaptations in response to specific selective pressures to cope with this challenge. To understand such biological adaptations we analyzed genome-wide SNP data under a Bayesian statistics framework, looking for outlier markers with an overly large extent of differentiation between populations living in a tropical forest, as compared to genetically related populations living outside the forest in Africa and the Americas. The most significant positive selection signals were found in genes related to lipid metabolism, the immune system, body development, and RNA Polymerase III transcription initiation. The results are discussed in the light of putative tropical forest selective pressures, namely food scarcity, high prevalence of pathogens, difficulty to move, and inefficient thermoregulation. Agreement between our results and previous studies on the pygmy phenotype, a putative prototype of forest adaptation, were found, suggesting that a few genetic regions previously described as associated with short stature may be evolving under similar positive selection in Africa and the Americas. In general, convergent evolution was less pervasive than local adaptation in one single continent, suggesting that Africans and Amerindians may have followed different routes to adapt to similar environmental selective pressures.

## Introduction

Tropical forests are characterized by a high diversity of plants, with tall trees, dense canopies and low light penetration [1]. Their climate is generally warm with minimum temperatures well above the freezing point and mean annual rainfall above 1,000 mm [2]. Despite being one of the most productive environments of the world, tropical forests provide only few resources for humans [3]. Indeed, in these environments plants invest most of their energy in structure maintenance and not into the reproductive organs that are the most edible parts for humans and their prey species [2]. In addition, the instability of food resources in response to the high seasonality of rainfall raises the costs of foraging, further reducing its capacity to support human life [2,3].

Besides food limitation, other characteristics of tropical forests may also contribute to the hostility of these environments. For instance, tropical areas harbor on average 70% higher human pathogen diversity as compared to more temperate areas [4]. As a consequence, infant and child mortality rates among tropical forest dwellers should be high [5]. Moreover, the small differences between air and skin relative humidities and high temperature, coupled with little air movement, make sweat production and evaporation difficult in tropical forests, potentially compromising thermoregulation [6].

Due to the hostility of this environment, it is unclear whether humans have ever subsisted in tropical forests without depending on external resources, such as agriculture or possible exchanges with neighboring populations. Evidence of societies living in such harsh conditions is scarce for contemporary modern humans [2], as well as for early *Homo* [7]. Nonetheless, it is possible that humans have developed recent biological adaptations to tropical forests. A few examples of such adaptations have indeed been documented, the most well-known being the pygmy phenotype, defined by Perry and Dominy [6] as small human body size (mean adult male height < 155 cm). These authors argue that short-statured individuals may have advantages to cope with food limitation, thermoregulation, and mobility hardship in a dense forest and, with few exceptions, are thus found in hunter-gatherer populations living in tropical rainforests of Africa, Asia, Oceania, and the Americas [6]. However, it has also been suggested that this phenotype could be a by-product of selection for early onset of reproduction [8], which could enable populations to overcome problems related to their life history and increased mortality [9].

To investigate whether tropical forest dwellers have developed specific biological adaptations to this harsh environment, we searched for genome-wide signals of positive selection in populations from the Americas and Africa, specifically aiming at identifying convergent evolution signals, that is a significant signal of positive selection occurring at the same genomic region or biological pathway in populations belonging to two distinct evolutionary lineages. To that effect, we investigated populations living in tropical forests and others, genetically related, living outside these environments using publicly available genome-wide SNP data and a robust and sensitive F_ST_-based method for inference of positive selection that explicitly includes a convergent selection model.

## Subjects and Methods

### Populations and samples

Genome-wide single nucleotide polymorphism (SNP) data were downloaded for seven populations included in the Human Genetic Diversity Panel database (HGDP; [10, 11]). Two African (Biaka (n = 30) and Mbuti pygmies (n = 15)) and two American (Surui (n = 21) and Karitiana (n = 24)) tropical forest populations were selected, as well as three other populations from these continents to serve as non-tropical forest comparisons (Mandenka (n = 24) and Yoruba (n = 24) in Africa; Pima (n = 25) in America). Considering the genetic similarity between Mandenka and Yoruba [12], these populations were grouped into a single set, hereafter called “West Africa”, to increase sample size and the statistical power of the analyses. More information on the chosen populations can be found in [3, 10–14] and S1 Table. We excluded atypical and duplicated samples, keeping only those present in the H1048 subset of [15].

The six populations were combined into four distinct population sets (PS), each including one tropical forest and one control population per continent as follows:

- PS1: West Africa, Biaka; Pima, Surui.
- PS2: West Africa, Mbuti; Pima, Surui.
- PS3: West Africa, Biaka; Pima, Karitiana.
- PS4: West Africa, Mbuti; Pima, Karitiana.

Each PS was analyzed separately to find loci and genomic regions that would putatively be under natural selection. The rationale behind the use of different population sets is to look for congruent signals of adaptation across data sets, and thus to eliminate signals potentially due to particular tropical forest populations, which have been shown to present high rates of genetic drift due to their small effective population sizes [16, 17].

The results of this analysis are the core of this study, but since we had a single non-forest dwelling Amerindian population, we performed a supplementary analysis with a similar sampling design as described above, except that we replaced the Mexican Pima by the Zapotec. The rationale behind this approach is to check whether the signal observed using the core analyses is not related to any adaptive evolutionary phenomenon that would be restricted to Pima (non-forest) rather than to the forest dwellers Karitiana and Surui. Thus, by replacing Pima by Zapotec—another non-forest agriculturalist population—we should be able to check if the reported signals hold. This analysis is performed separately, because the number of shared SNP for this dataset (364, 470 SNPs) is much lower than that of the HGDP database (660,918 SNPs). We therefore studied the following supplementary population sets (SPSs):

- SPS1: West Africa, Biaka; Zapotec, Surui.
- SPS2: West Africa, Mbuti; Zapotec, Surui.
- SPS3: West Africa, Biaka; Zapotec, Karitiana.
- SPS4: West Africa, Mbuti; Zapotec, Karitiana.

### Genetic data

Data on 660,918 SNPs (Illumina HumanHap 650K Beadchips) were downloaded from the Stanford University HGDP-CEPH SNP genotyping data supplement 1 ([12]; <ftp://ftp.cephb.fr/hgdp_supp1/>). We initially discarded 250 markers that were monomorphic in all populations, those that presented only missing data, and those located on either the Y-chromosome (due to low SNP density in the chip), the pseudoautosomal region of the sex chromosomes, or the mtDNA, leaving us with 660,668 SNPs. Within each PS defined above, those markers with minor allele frequency below 5% in all populations joined into one were discarded, yielding 582,074, 581,855, 584,205, and 577,345 SNPs for population sets PS1–PS4, respectively.

The supplementary dataset includes merged data from seven different sources, [18]. The Zapotec sample was genotyped on Illumina HumanHap 550 V3.0 arrays obtained partially from MGDP [19] and partially from samples newly genotyped by Reich et al. [18]. Surui data includes samples genotyped by HGDP [10, 11] and Kidd Lab [18] on Illumina 650Y arrays. Data for the remaining populations are from the same source as the core analysis, i.e. HGDP [10, 11]. This merged dataset was compiled and processed according to stringent data curation and validation procedures. For our study, we used data on segments of potentially non-native origin that were originally masked by Reich et al. [18] with a local ancestry inference software. This yielded a sample size of 43 individuals for Zapotec (merging Zapotec1 and Zapotec2), 13 for Karitiana and 24 to Surui. Readers are referred to the source publications for further details of genotyping, masking, and filtering [18, 19].

### Detecting outlier SNPs

A modified version of BayeScan [20, 21] was used to identify candidate targets for natural selection. The original methodology in this software is based on the multinomial-Dirichlet likelihood-based approach [22] implemented via a Markov chain Monte Carlo (MCMC) algorithm [23]. The approach assumes an island model [24]—in which the subpopulations’ allele frequencies are correlated through a common migrant gene pool from which they differ by varying degrees—to calculate a population-specific F_ST_ coefficient. Logistically transformed F_ST_ coefficients are then decomposed into a population-specific component (β), shared by all loci, and a locus-specific component (α), shared by all the populations [20, 21, 23]. Selection is inferred when α is significantly different from zero. For each locus, two alternative evolutionary models including α (selection) or neutrality can thus be explored. The posterior probability of each model (selection vs. neutrality) is estimated with a reversible-jump MCMC algorithm [20, 21] and indicates how likely the model with selection is in comparison to the neutral one. Significantly positive values of α are indicators of an overly large level of differentiation of a given SNP, which could be either due to positive or to balancing selection in a given environment. Alternatively, significantly negative values of α are indicative of simultaneous or global balancing selection in the two environments, maintaining allele frequencies to similar levels in all populations. Note that the method has limited power to detect balancing selection with only two pairs of populations, a limitation not associated to the inference of positive directional selection [20, 21]. Thus with the study design proposed here, it is possible that an apparent lack of instances of global balancing selection may be actually due to a high false-negative rate for significant negative α values. Further information on this methodology can be found in the BayeScan manual or other methodological papers on the F-model [20–23].

The latest version of BayeScan [21] includes a hierarchical island model accounting for the relatively closer similarity of certain populations in comparison to others, as should be the case for populations that are sampled in a given continent and therefore share part of their history. According to the hierarchical island model, each continent has a specific migrant pool. An F_SC_ coefficient measures the differentiation of each population within the continental pool of migrants and an F_CT_ coefficient measures the differentiation of each continent within the overall meta-population [21]. In this regard, when considering two pairs of populations in two different continents, one ends up with four alternative selection models for each locus: (1) neutral variability; (2) selection in one continent; (3) selection in the other continent; and (4) selection in both continents. BayeScan estimates for each marker the posterior probability of each of the four tested models. These posterior probabilities are then transformed into *q*-values for each marker in order to control for the False Discovery Rate (FDR; [25]) considering the probability of a SNP being under selection regardless of which model (selection in one or both continents). FDR is defined as the expected proportion of false positives among outlier markers. Further details on the hierarchical BayeScan methods, its power and sensitivity can be found in ref [21].

For an F_ST_-based method such as the one implemented in Bayescan, the levels of genetic differentiation between the analyzed populations and sample sizes are crucial for the power to detect natural selection. The populations analyzed in this study come from two opposite extremes of genetic diversity observed in humans [16–18] and Bayescan was shown to have low power to infer global balancing selection in a scenario with low genetic differentiation or directional positive selection with a small number of sampled populations with high genetic differentiation [20]. In this paper, we exclusively discuss the results generated for the posterior probabilities for each loci being targets of natural selection inside each continent (an estimate based on F_SC_) and disregard the results obtained for the highest hierarchical level (i.e. between continents; F_CT_), which could be due to other environmental differences between these two continents. By doing so, we still take into account the divergence between the two continents, but we avoid any particular bias or power decrease that would result in ignoring them [21]. As for the effect of sample size upon statistical power, Bayescan was shown to perform well with as few as 15 individuals per population [20], which is smaller than the sample sizes considered for this study.

Alternatives to F_ST_-based natural selection tests are those based on Linkage Disequilibrium (LD). Those tests use information on homozygosity in single populations, which makes it difficult to formally test for differences in levels of LD and the length of homozygosity tracts over all populations. This is precisely the strength of the current approach, the integration of all populations in a single statistical analysis. A detailed comparison of this approach to other available methods can be found in ref [21].

In this analysis, we considered all SNPs with *q*-values lower than 0.1 as potentially significant. This procedure yielded a list of outlier SNPs for each PS, which were then considered as candidate loci for natural selection targets. We finally assume hereafter that convergent selection occurred when selection presents a higher probability than neutrality and model 4 has a higher posterior probability than models 2 and 3.

### Gene and regulatory elements annotation

All SNPs were assigned to genes using PLINK v1.07 ([26]; available at http://pngu.mgh.harvard.edu/purcell/plink). SNPs not present in coding regions were assigned to a given gene if located less than 50 kb away from it. When more than one gene was within this range, the closest gene was chosen for the subsequent analyses. This software was also used for indicating the attributes of the functional SNPs, i.e. if they are nonsense (stop codon), missense (non-synonymous), frameshift or splice-site mutations, based on information from dbSNP build 129 available with PLINK v1.07. The amount of outlier SNPs falling in genic regions (or < 50 kb away from them) was compared to the amount of outlier SNPs in non-genic regions first taking into account only protein coding genes and then all regulatory elements as described below. This proportion was then compared to the distribution of the 660,668 HGDP SNPs in genic or non-genic regions with a Fisher exact test using R [27] to check if these outlier SNPs were enriched for genic SNPs in comparison to all available markers. This test assumes that SNPs are independent from each other, which may not be the case for all markers. To control for a possible bias due to LD, we repeated the test (N = 1000) by sampling randomly one outlier SNP and one non-outlier SNP per 100 kb window.

We tested whether the number of outlier SNPs and genes shared among the population sets was higher than expected. For this we repeatedly (*N* = 50,000) sampled randomly the same number of SNPs as the counts of outlier SNPs from the population sets PS1-PS4 and counted the number of overlapping SNPs. We assessed the p-value by taking the proportion of occasions that resulted in an overlap equal or larger than the overlap we found. We followed the same procedure when testing the overlap of outlier genes.

The hg19 assembly coordinates (NCBI Build 37.3) of 19,683 protein-coding genes located on the human autosomal and X chromosomes were obtained from the NCBI Entrez Gene website ([28]; <http://www.ncbi.nlm.nih.gov/gene>, accessed on February 7, 2013). Seventeen genes were annotated with multiple locations (caused by merging of gene records in the NCBI database); in these cases we took the outermost start and end positions.

The original positions of the SNPs on the hg18 reference genome (NCBI Built 36.3) obtained from the original dataset ([26]; <ftp://ftp.cephb.fr/hgdp_supp1/>) were remapped on hg19 with the NCBI Genome Remapping Service (<http://www.ncbi.nlm.nih.gov/genome/tools/remap>). In doing so, we were not able to remap 74 SNPs, which were then excluded from the following analyses.

Information on the DNaseI Hypersensivity sites were obtained online (<http://hgdownload.cse.ucsc.edu/goldenPath/hg19/encodeDCC/wgEncodeRegDnaseClustered/>, volume 2, accessed on April 4, 2013) for the hg19 assembly and added to the annotation file. These clusters show DNaseI hypersensitive areas assayed in 125 human cell types by the ENCODE Project [29] and may be indicative of regulatory regions. This information was used to calculate the distance of an outlier SNP to a putative functional region.

### Detecting clusters of outlier SNPs

A sliding-window approach was implemented in R to identify significant clusters of candidate SNPs and remove isolated loci. We considered consecutive windows of size 500 kb wide with a shifting increment of 25 kb at each step. Despite the acknowledged differences in the extent of LD in the analyzed populations due to different time-scales and other particularities of the demographic history of the continent they are located [16–18], a single window size was considered here in order to normalize the analysis. By doing so, there is a chance to include false negatives in those populations where recombination was more frequent. The *q*-value associated to each window was assumed as the 95% quantile of the *q*-values calculated for each SNP included in the window in order to avoid windows with one outlier SNP to be set as significant and to account for a potential SNP density bias. Those 500 Kb windows with a SNP density less than 20% of their chromosome average were set at non-significant. A graphical representation of this procedure (Manhattan plots) was plotted with R taking into account the physical position of the SNPs, their particular *q*-value, the window *q*-value, and the best supported model of selection (if in Africa, the Americas, or both continents) for each outlier SNPs. For the outlier SNPs, we also included information on their nearest gene if it was at most 50 kb apart.

The sliding-window approach yielded a second set of outlier markers. This SNP set is more refined than the one with the first candidates, since it ignores the low SNP density regions of the genome and those candidates that are isolated, highlighting genomic regions with a higher density of outlier SNP.

### Gene set analysis

We applied a gene set enrichment analysis as described by Daub et al. [30] to find pathway level signals of selection. In short, this method tests whether genes in a gene set show a shift in the distribution of a certain selection score. In our case, we took as selection score the probability for selection in Africa (*p*_*af*_), the Americas (*p*_*am*_), and both continents (*p*_*co*_, convergent selection). We also added the score *p*_*sl*_, which is the probability of any selection (*p*_*sl*_ = *p*_*af*_ + *p*_*am*_ +*p*_*co*_), which means selection in only one or both continents. As we use one value per gene in the enrichment test, we transformed the SNP-based scores to gene-based scores. First, SNPs were assigned to genes as described above: a SNP was assigned to a gene if the SNP location was within a gene transcript; otherwise it was assigned to the closest gene within 50 kb distance. For each gene and each selection model, we took the highest selection score of all SNPs assigned to this gene. After removing 1,922 genes with no SNPs assigned, a list of 17,761 genes remained for PS1 and PS4, 17,766 for PS2, and 17,769 for PS3.

We collected 2,336 gene sets from the NCBI Biosystems database ([31], http://www.ncbi.nlm.nih.gov/biosystems, downloaded 3 Sep 2013). After removing genes that were not part of the gene list mentioned above, excluding gene sets containing less than 10 genes and pooling (nearly) identical gene sets into union sets, 1,216 (PS1 and PS2) and 1,217 (PS3 and PS4) sets remained and they were used as input in the enrichment tests.

We computed the SUMSTAT [32] score for each gene set, which is the sum of either the *p*_*af*_, *p*_*am*,_ *p*_*co*,_ or *p*_*sl*_ values of all genes in a set, depending on the selection model. Potential candidates for selection are gene sets that score a high SUMSTAT value. To assess significance, we compared the SUMSTAT score of each tested gene set with a null distribution created from random gene sets of equal size. To improve computation time, the creation of the null distribution was done with a sequential random sampling method. We first tested all sets against 10,000 randomizations. Next, for each tested gene set, we counted the number of random sets with the same or higher SUMSTAT score. Only for those sets with a count smaller than 5,000, we expanded the null distribution with another 10,000 randomizations. This process was continued until we reached a maximum of 500,000 randomizations.

Selecting the highest score among the SNPs in or near a gene can induce a bias, as it is more likely that genes with high SNP density have an extreme value assigned. We corrected for this potential bias by placing genes in bins of similar SNP densities and constructing the null distribution from random gene sets with the same bin distribution as the gene set being tested. Note that we could not rescale the gene scores per bin as was done in [30], because the gene scores per bin follow a skewed distribution and rescaling would lead to a potential loss of the signal.

We removed the redundancy among candidate gene sets by applying a 'pruning' method according to which we iteratively assigned shared genes between sets to the highest scoring gene set. Thus, we gain more insight into which gene sets actually give the significant signal. As these tests were not independent anymore, we estimated the q-value of these pruned sets empirically. All sets scoring a q-value below 20% (before and after pruning) were reported.

We used *R* [27] and Cytoscape [33] to visualize the significant pathways and their overlap using a layout that was inspired by the Cytoscape plugin EnrichmentMap [34].

## Results

According to the F_ST_-based Bayesian approach implemented via BayeScan [20, 21] 1,482, 1,222, 1,579, and 1,365 outlier SNPs were identified in populations sets PS1 to PS4 respectively, ranging from 0.21 to 0.27% of the SNPs analyzed. From these, 75 SNPs are significant regardless of which PS is analyzed. There were no cases in which balancing selection could be inferred, i.e. α never reached a significant negative value, which may be due to the characteristics of the genetic system employed (e.g. low polymorphism [20] or a possible ascertainment bias), to the lack of power to detect markers under balancing selection [23], or to the low number of populations analyzed [20], but not necessarily to the absence of this phenomenon in the evolutionary history of these populations.

Using a threshold of 50 kb, 568, 474, 620, and 517 genes were associated with those significant SNPs from PS1 to PS4 respectively, of which 57 genes were found to be co-occurring in all four PSs (namely *ABLIM3*, *ACSS2*, *AKAP6*, *ANKRD26*, *ARHGEF10*, *ATIC*, *BCAT1*, C20orf111, C2orf73, *CBLN1*, *CNTN4*, *CNTNAP5*, *COL22A1*, *CPA5*, *CRTC3*, *CWH43*, *DCUN1D4*, *DHCR7*, *EPHB4*, *FAM188B*, *FKBP6*, *GALNT16*, *GLIS3*, *GLRB*, *GPC6*, *GRIK2*, *HLA-DPA1*, *HMG20B*, *HSF2*, *IQGAP1*, *KIAA1598*, *KLHL29*, *LRRC66*, *MASTL*, *MPST*, *NAALADL2*, *NRG1*, *NRP2*, *NSUN5*, *PARK2*, *PKIB*, *PPP2R2C*, *RABGGTB*, *RAD51B*, *RBFOX1*, *RBM9*, *RBMS3*, *RFX3*, *ROBO2*, *SCP2*, *SGCB*, *SHISA6*, *SPAG16*, *SPATA13*, *SPATA18*, *ST6GAL1*, and *TRG@)*. We confirmed with a random permutation test (N = 50,000) that the number of outlier SNPs (75) and genes (57) shared by all population sets is higher than expected by chance (p-value < 2e-5).

The sliding-window procedure yielded 440, 399, 505, and 471 SNPs with a significant signal of positive selection for each PS (S1 File). From these, 19 are found to be functional non-synonymous mutations (missense) in one or two PSs (S2 Table). There was no instance of a significant SNP defined as nonsense, frameshift, or splice-site mutation.

Outlier SNPs are significantly enriched (Fisher exact test, p-value < 0.022) for genic SNPs in comparison to the whole HGDP SNP set. On average 72.8% of the outlier SNPs after the sliding-window procedure are located in or less than 50 kb away from protein-coding regions considering all four PSs, while for all SNPs in the HGDP database this proportion is only 63.6%. This enrichment is not due to potential LD between outlier SNPs, as it still holds when randomly sampling one outlier SNP and one non-outlier SNP per 100 kb window (N = 1000, mean p-value = 0.02, 90% CI [2.3e-4, 9.1e-2]). Moreover, all outliers were located at maximum 45.8 kb apart from a gene or from a putative regulatory element indicated by a hypersensibility to DNaseI.

**Fig 1:**
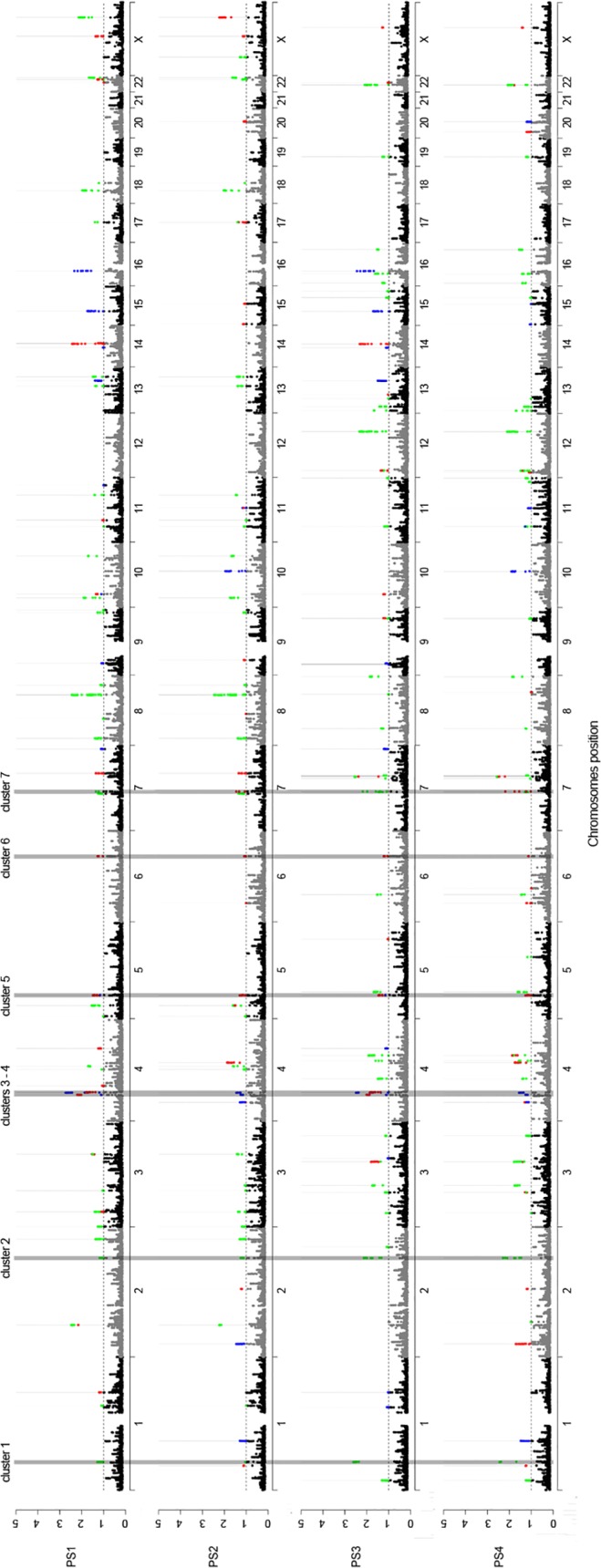
Manhattan plots of the physical position of SNPs (*x*-axis) and corresponding *q*-values in log10 scale (*y*-axis) for inferring selection. Based on a sliding-window approach, SNPs are color-coded according to the best supported model of selection, namely neutrality (black or grey), in Africa (blue), in the Americas (green), and in both continents (convergent evolution, red) with a False Discovery Rate of 0.1 (dashed line). Different sets of populations were used in the analysis yielding four different population sets (PS1–4). Congruent clusters of outlier SNPs considering all four PSs are highlighted with a grey box.

Fig. 1 shows the Manhattan plots of the physical position of the SNPs in the genome and their estimated *q*-values for the different PSs, as well as the best model for selection (color-coded) in the sliding-window approach. It was possible to identify seven clusters of outlier SNPs co-occurring in all four PSs (indicated by gray-shaded boxes at Fig. 1 and at S1–S6 Figs.). Their coordinates, size, and genic content are described in Table 1.

**Table 1:** Coordinates, size, and genic content of the seven clusters of outlier SNPs occurring in all four population sets (PSs) considered in the analysis.

Cluster	Coordinates in hg19	Size	SNPs^a^	Genes^b^
1	chr1:53,476,720–53,520,376	43.7 kb	rs6679819, rs10437066, rs6588459, rs7550236	*SCP2*
2	chr2:184,608,065–184,633,769	25.7 kb	rs17715017, rs1733497, rs1439771, rs2119047	-
3	chr4:48,960,613–48,972,901	12.3 kb	rs2605267, rs2572363	*CWH43*
4	chr4:52,758,044–52,935,931	117.9 kb	rs4865414, rs1519590, rs178724, rs1460554	*DCUN1D4*, *LRRC66*, *SGCB*, *SPATA18*
5	chr5:43,497,655–43,862,944^c^	365.3 kb	rs10062920, rs4449542, rs7721405, rs6875400	*CCL28*, C5orf34, *NNT*
6^d^	chr6:122,738,019–122,869,764	131.7 kb	-	*HSF2*, *SERINC1*, *PKIB*
7	chr7:72,722,731–72,750,595	27.9 kb	rs1880948, rs1178970	*NSUN5*, *FKBP6*

^a^Outlier SNPs co-occurring in all PSs.

^b^Nearest genes located at least 50 kb away from an outlier SNP in at least two PSs.

^c^For PSs 2 and 4, cluster 5 is defined more broadly, starting at position 43,416,999, including rs4264950 and rs7720858.

^d^No outlier SNP at this cluster co-occurred in all four PSs, but in each case four outlier SNPs were found (rs2816141, rs1741820, rs487098, and rs197686 for PSs 1 and 3; and rs3778348, rs3778348, rs9490478, rs9320878 for PSs 2 and 4).

For the first two clusters, the most likely model of selection for the majority of outlier SNPs is positive selection in the Americas; however, for a few SNPs convergent evolution cannot be ruled out (Fig. 1). While the first cluster includes the gene *SCP2* (S1 Fig.), the second cluster lies in an inter-genic region (S2 Fig.). The next two clusters of significant SNPs occur on the same chromosome and selection in Africa is the best-supported model in most cases, although convergent selection is likely for a few SNPs (S3 Fig.). Cluster 3 is associated with gene *CWH43*, while cluster 4 includes SNPs that fall into genes such as *DCUN1D4*, *LRRC66*, *SGCB*, and *SPATA18* and their vicinity. Convergent evolution is the best-supported model of selection for the majority of SNPs of the remaining three clusters (S4–S6 Figs.). Cluster 5 is associated with the following three genes: *CCL28*, *C5orf34*, and *NNT;* which may or may not present a significant signal of selection depending on the analyzed PS (S4 Fig.). Cluster 6 includes genes *HSF2* and *PKIB* for all PSs, and the analysis of PS1 and PS3 revealed an additional significant SNP located at *SERINC1*. The last cluster (no. 7) includes two SNPs, one located in a *FKBP6* intron and the other located 0.12 kb apart from *NSUN5* and 3.55 kb from *TRIM50*. The abovementioned 14 genes that include or are close (<50 kb) to SNPs that present signals of positive selection in the sliding-windows approach for all four PSs are described in Table 2.

**Table 2:** Genes with signals of positive selection suggesting human adaptations to tropical forests in Africa and the Americas.

Gene	Cluster	Biological function in mammalians or associated human diseases
*SCP2*	1	Involved in cholesterol trafficking and metabolism [47].
*CWH43*	3	Enhance lipid remodeling to ceramides [48].
*DCUN1D4*	4	Unknown.
*LRRC66*	4	Unknown.
*SGCB and SPATA18*	4	Is located in a genomic region where a microdeletion causes Limb-girdle muscular dystrophy type 2E with joint hyperlaxity and contractures [42].
C5orf34	5	Unknown.
*CCL28*	5	Modulate immunity to viral infection [49] and skin-related inflammatory diseases [50].
*NNT*	5	Produces high concentrations of NADPH at mitochondria and the resulting energy is used for biosynthesis and in free-radical detoxification [51].
*HSF2*	6	Involved in the activation of heat-shock response genes under conditions of heat [52].
*PKIB*	6	Associated to the aggressive phenotype of prostate cancer [53].
*SERINC1*	6	Unknown.
*FKBP6*	7	May play a role in modifying the susceptibility to idiopathic spermatogenic impairment [54].
*NSUN5*	7	Deleted in Williams-Beuren syndrome (vascular system and calcium metabolism problems) [55].
*TRIM50*	7	May be involved in the Williams-Beuren syndrome [56].

Five of the seven clusters showing signs of positive selection with the HGDP data sets also show signals of positive selection in the supplementary analysis. The exceptions are cluster 3 on chromosome 4, which was not covered by any SNP in the smaller data set, and cluster 7 on chromosome 7, for which the signal of positive selection disappears after replacing Pima by Zapotec. In the supplementary analysis, no additional clusters with signal of positive selection were found in all SPSs. The type of natural selection—convergent evolution or selection in a single continent—inferred from the supplementary data set is generally concordant with the analysis done on HGDP data, except for cluster 6 on chromosome 6, where selection in Africa is more important in the supplementary analysis. After performing the sliding-window approach, cluster 1 appears for SPS1 and SPS3, clusters 4 and 5 for all SPSs, and cluster 6 for SPS2 and SPS4.

Fifteen genomic regions had been found to be associated with the pygmy phenotype by means of covariation between allele frequencies and body height in Africa in another study [35]. We found signals of positive selection in the African continent in four of these regions. By comparing Mbuti to West Africa, Mendizabal et al. [35] identified two genomic regions as candidate for selection. One of them, located in the long arm of chromosome 10, is among those with the highest *q*-values in our study (Fig. 1). It includes 14 outlier SNPs in the following 11 genes: *P4HA1*, *NUDT13*, *ECD*, *FAM149B1*, *DNAJC9*, *MRPS16*, *ANXA7*, *ZMYND17*, *USP54*, *PPP3CB*, and *TTC18*. Two outlier SNPs in this region—rs2271904 and rs4294502—are both non-synonymous mutations in *ECD* and *TTC18* respectively. The second region is defined by two SNPs (rs7174731 and rs7181518) in *TRIP4*. In a third region, we found two genes with positive selection signals considering West Africans in comparison to Biaka instead of Mbuti: *USP46* and *MLL3*. An additional region suggested by Mendizabal et al. [35] to be associated with the pygmy phenotype presents signals of convergent adaptation using all four different combinations of populations in our study. They found significant SNPs in *NNT*, while we observed a more diffuse significant signal also including *CCL28* in some cases (S4 Fig.).

**Fig 2:**
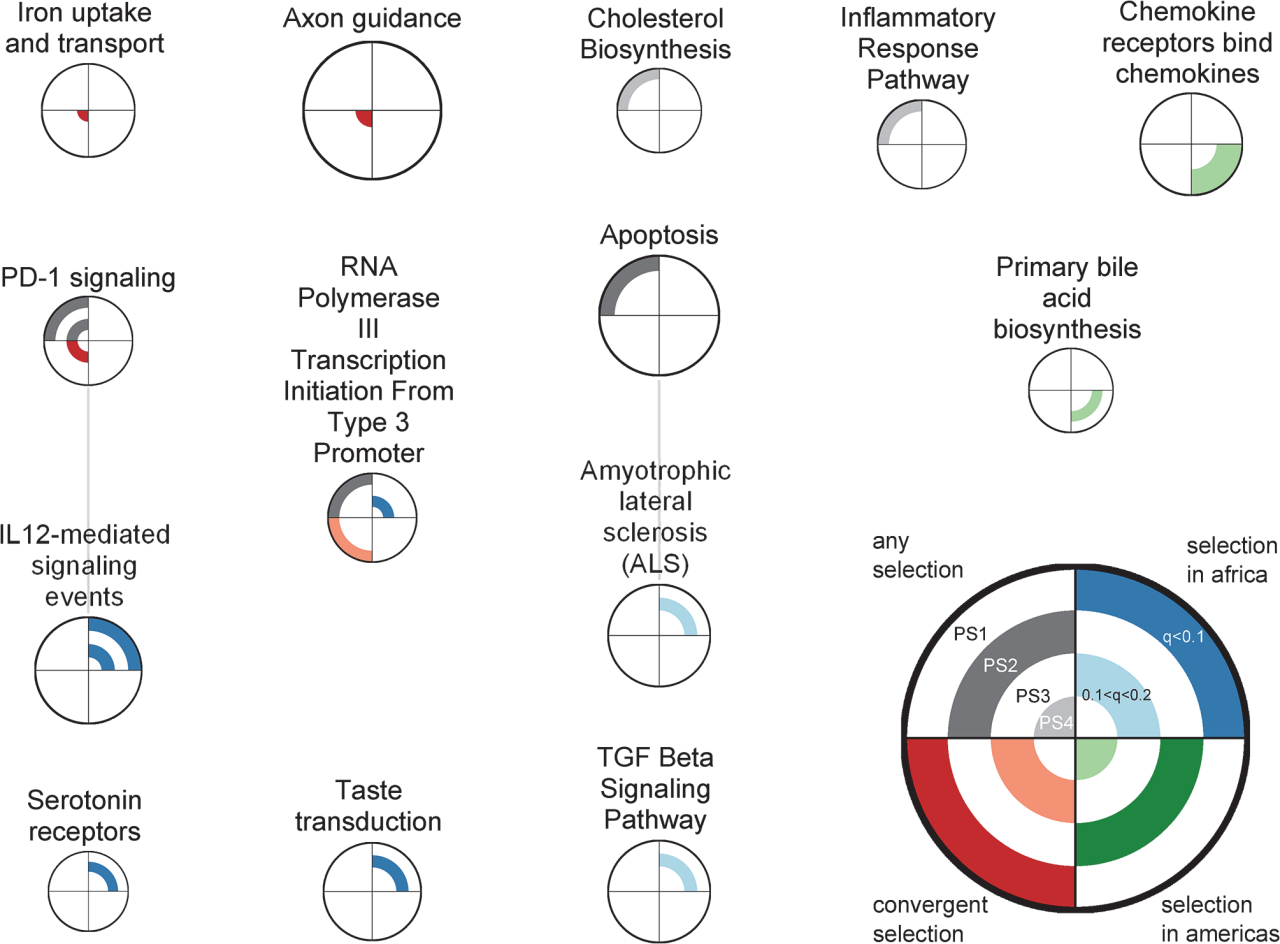
Gene sets significantly enriched for selection signals. Nodes are gene sets that score a q-value ≤ 20% after pruning in the gene set enrichment test (See S7 Fig. for the equivalent plot regarding the results before pruning). Gene sets are connected when at least one of them shares >33% of its genes with the other set before pruning. The size of a node scales with the size of the gene set. Each quadrant of a node represents results on one of the four selection scores, while the four rings in a node correspond to the four population sets (different combination of populations; see Methods). The color of each quarter of a ring corresponds to the significance of the test result (dark colored: q≤10%, light colored: 10%<q≤20%, white: q>20%).

As selection is acting on phenotypes instead of genotypes, we applied a gene set analysis [30] to detect pathways involved in adaption to living in tropical forests. We found 43 pathways scoring a q-value below 20% for at least one of the population sets and selection models (S7 Fig.), most of them scoring significant for selection in Africa. Among those we find a large cluster of 22 gene sets involved in immune response, nine of which show signals of convergent selection, and a cluster of 11 gene sets involved in RNA Polymerase III transcription initiation. In order to eliminate redundancy, we assigned overlapping genes to the highest scoring pathway. After this ‘pruning’ procedure, 14 pathways remain significant, including “Apoptosis” and “Cholesterol Biosynthesis” among other immune and nervous-system related pathways (Fig. 2). Four of these pathways present a consistent signal of positive selection across different PSs or selection models, namely “PD1-signaling”, “IL 12-mediated signaling events”, “RNA Polymerase III Transcription Initiation From Type 3 Promoter”, and “Chemokine receptors bind chemokines”. Note that taking a *q*-value threshold of 0.1 would still result in 8 significant gene sets, of which 3 show multiple signals of adaptation (data not shown).

## Discussion

Tropical forests are believed to be very harsh habitats for human beings [3]. In addition to being almost deprived from energy-rich food and edible plants [2], these environments are very propitious for the development of diseases [4] and might also compromise thermoregulation [6]. In this work we used a Bayesian method [20, 21] to identify SNPs in a genome-wide dataset showing overly large or low extent of differentiation between tropical and non-tropical forest populations. We sought to infer signals of convergent evolution by comparing native populations from tropical forests (Biaka, Mbuti, Karitiana, and Surui) with genetically related populations living elsewhere (Mandenka, Yoruba, and Pima) combined in four different population sets (PSs). The analysis suggested some SNPs, genes, and a few biological pathways in which convergence could be inferred. These outlier SNPs are found to be enriched for genic loci even after correcting for a potential LD bias. Those few cases that fall within an intergenic region, such as Cluster 2 (S1 Fig.), could always be associated (distance < 50 kb) to a putative regulatory element described by the ENCODE project. These results suggest that regulatory regions could also be involved in some recent human adaptations [36]. Genomic regions where the same signal of selection could be identified by employing different combinations of populations (clusters 1–7 on Fig. 1) suggest that the inferred signal is due to environmental selective pressures and adaptation rather than to the demographic history of the populations and those regions are the main focus of our discussion. For five out of the six clusters with sufficient SNP coverage in the smaller supplementary dataset, we observed significant SNPs with the same type of selection—convergence or selection in one continent—as observed in the analysis of the HGDP data. The exception is cluster 7 where the signal of convergent adaptation does not hold when we replace Pima by Zapotec. In this case, selection in Africa is the best-supported model. Based on the agreement between results generated with the two datasets, results for clusters 1, 2, and 4–6 should thus be considered as very robust, but results obtained for cluster 7 should be considered with some caution. We can see two reasons for an absence of congruent results for cluster 7. First, it could be that our supplementary analysis did not have enough power to detect signal given the lower number of SNPs available in Zapotec. Else, our initial results for cluster 7 could be due to the own evolutionary history of the Pima and have nothing to do with adaptation to tropical forests. This could be due to an environmental pressure shared between West African populations and Pima, but not present in Zapotec. It appears difficult to distinguish between these hypotheses without having more samples genotyped for a larger number of SNPs.

In a previous study involving Native Americans, Hünemeier et al. [37] suggested that *ABCA1*, a gene encoding a cholesterol efflux regulatory protein, was evolving under positive selection due to limited food resources that Native Americans encountered during their history. We also found significant signals of positive selection in genes that are related to lipid circulation and metabolism, such as *SCP2* and *CWH43* (Table 1) and in a genetic pathway related to cholesterol biosynthesis (Fig. 2 and S7 Fig.). Besides playing a role in nutrition, these genes could also be involved in immunological response, since cholesterol plays an important role in various infectious processes such as virus invasion and replication [38] as well as in resistance against malaria [39].

In this regard, Sabeti et al. [40] already noticed a preponderance of genes related to the immune system in available genome-wide scans for positive selection. This prevalence was further confirmed by Williamson et al. [41], López Herráez et al. [42], and Daub et al. [30]. Besides the two genes mentioned above—*SCP2* and *CWH43*, which have a possible role in immunity—the protein encoded by *CCL28* modulates immunity to HIV infection and skin-related inflammatory diseases. Additionally, the selective pressure of this class of genes may also be important at the multi-genic level, since a network of 22 pathways involved in immune response presents signals of positive selection (S7 Fig.). After pruning, two of these pathways remain significant for more than one PS, namely “PD-1 signaling” and “IL 12-mediated signaling events”. This indicates that they both have independently a strong selective signal, which would deserve further investigations for their role in adaptations to tropical environments.

Another category of genes that frequently presents signals of positive selection is fertility, more specifically, spermatozoid development [40]. In this regard, *FKBP6*, a male fertility factor, is found here to be potentially evolving under positive selection in our analyses (S6 Fig.), but this observation has to be taken with caution as this gene is not identified as an outlier in our supplementary analysis when Ameridian forest populations are compared to Zapotec instead of Pima.

Heat-shock transcription factors, such as that encoded by *HSF2* (Cluster 6; S5 Fig.), are activated by stress and respond to elevated temperatures. One of the consequences of inefficient thermoregulation is the increase of body temperature. The observed positive selection signals at SNPs found in this gene could be due to an adaptation to the tropics, initiating gene(s) transcription in response to high body temperatures. Another study with African-, European-American, and Chinese populations also found a significant signal of positive selection in heat shock genes [41], suggesting that this category might have some importance in human adaptation to different environments worldwide.

It is generally accepted that the pygmy phenotype might have evolved as an adaptation to life in dense tropical forests, to thermoregulation, and to food scarcity [6] or as a by-product of selection for early onset of reproduction [8]. Our research design enables the comparison of two African pygmy populations with two other non-pygmy populations from the same continent, from which inferences can be drawn about the differences in selective pressures that African pygmy and non-pygmy populations are subjected to. Clusters 3 and 4 (S3 Fig.) are the two main regions where we found signals for positive selection in Africa. The first cluster presents a gene involved in lipid metabolism (*CWH43*, discussed above) and the second includes the genes *DCUN1D4*, *LRRC66*, and *SGCB*, which are found in a region known to be associated to severe limb-girdle muscular Duchenne-like dystrophy [43].

Other regions of interest for the study of the pygmy phenotype are those four in which we found positive selection signals and are associated with the pygmy phenotype according to another study [35]. From the genes found in this region, three are notable for being also associated with height, bone development or the pygmy phenotype in previous studies. Those genes are *PPP3CB*, which encodes a subunit of calcineurin, a protein that regulates bone formation by osteoblast differentiation [44]; *TRIP4*, a gene with positive selection in pygmy populations [45] that relates to a category (thyroid hormone receptor) that was suggested to be associated with this phenotype [42]; and *MLL3*, involved in histone modification, a category associated with height in a genome-wide association study [46].

In general, non-convergent positive selection signals were more pervasive than signals of convergent evolution at the SNP, gene and pathway levels. This suggests that the similar selective pressures imposed by the tropical forest environment in Africa and the Americas have targeted different genes and pathways. This could be a result of different genetic background of the populations from these two continents and also a result of the differences in the time-scales that adaptation took place, since the Americas were peopled more recently than Africa [16–18]. As it is particularly true for the pygmy phenotype [45], putative adaptations to the tropical forests could be the result of selection acting on different genetic targets even though their result might be similar at the phenotypic level. Nonetheless, due to the power limitation associated with detecting convergent evolution [21], this model cannot be ruled out for those regions where at least one SNP was assigned to it among other SNPs assigned to a model of positive selection in one continent, which is the case of Clusters 1 to 4. Moreover, the scarcity of convergence examples could also be a result of the SNP density of the array employed, since the power to detect selection is determined by physical linkage between the sampled SNPs and the causal variant. In addition, due to different time-scales that such events took place, in populations such as the Africans linkage is more likely to be disrupted than in a younger population such as the Amerindians, which makes it necessary to use a denser SNP-array in order to rule out false-negatives in African populations. In other words, we might be missing some actual convergence signals due to a lack of sufficient linkage between the target region and the sampled SNPs in Africa and convergence might thus be underestimated in this continent. Therefore, it is likely that additional genes evolving under convergent evolution in these populations could be found by using different methods and genetic systems.

## Conclusions

The F_ST_-based hierarchical Bayesian method used in our study enabled us to detect a number of regions with positive selection, suggesting that the following biological functions and pathways may play a role in human adaptations to tropical forest: lipid metabolism, immunology, body development, and heat stress response. The same signals found in different population sets suggest that they are due to environmental adaptation rather than to the demographic history of the sampled populations. Further refinement of these analyses with e.g. full genome or exome sequence information could reveal which particular mutations are responsible for these adaptations. Moreover, the few cases in which convergent evolution could be inferred contrast with the larger amount of genes with non-convergent positive selection signals, suggesting that Africans and Amerindians may have followed different routes to adapt to similar environmental selective pressures.

## Supporting Information

S1 Fig Manhattan plots of the physical position of SNPs (*x-*axis) and their correspondent *q*-values (log-transformed at *y*-axis) for inferring positive selection in Chromosome 1. The sliding-window *q*-value is indicated by a yellow continuous line. With a False Discovery Rate of 0.1 (dashed line), SNPs are color-coded according to the best supported model of selection, namely neutrality (black), selection in Africa (blue), selection in the Americas (green), and in both continents (convergent evolution, red). When an outlier SNP was located less than 50 kb apart from a gene, the closest gene name was written next to it. Different sets of populations were used in the analysis yielding four different population sets (PS1: 1A; PS2: 1B; PS3: 1C; PS4: 1D).
(TIFF)

S2 Fig Manhattan plots of the physical position of SNPs (*x-*axis) and their correspondent *q*-values (log-transformed at *y*-axis) for inferring positive selection in Chromosome 2. The sliding-window *q*-value is indicated by a yellow continuous line. With a False Discovery Rate of 0.1 (dashed line), SNPs are color-coded according to the best supported model of selection, namely neutrality (black), selection in Africa (blue), selection in the Americas (green), and in both continents (convergent evolution, red). When an outlier SNP was located less than 50 kb apart from a gene, the closest gene name was written next to it. Different sets of populations were used in the analysis yielding four different population sets (PS1: 2A; PS2: 2B; PS3: 2C; PS4: 2D).
(TIFF)

S3 Fig Manhattan plots of the physical position of SNPs (*x-*axis) and their correspondent *q*-values (log-transformed at *y*-axis) for inferring positive selection in Chromosome 4. The sliding-window *q*-value is indicated by a yellow continuous line. With a False Discovery Rate of 0.1 (dashed line), SNPs are color-coded according to the best supported model of selection, namely neutrality (black), selection in Africa (blue), selection in the Americas (green), and in both continents (convergent evolution, red). When an outlier SNP was located less than 50 kb apart from a gene, the closest gene name was written next to it. Different sets of populations were used in the analysis yielding four different population sets (PS1: 3A; PS2: 3B; PS3: 3C; PS4: 3D).
(TIFF)

S4 Fig Manhattan plots of the physical position of SNPs (*x-*axis) and their correspondent *q*-values (log-transformed at *y*-axis) for inferring positive selection in Chromosome 5. The sliding-window *q*-value is indicated by a yellow continuous line. With a False Discovery Rate of 0.1 (dashed line), SNPs are color-coded according to the best supported model of selection, namely neutrality (black), selection in Africa (blue), selection in the Americas (green), and in both continents (convergent evolution, red). When an outlier SNP was located less than 50 kb apart from a gene, the closest gene name was written next to it. Different sets of populations were used in the analysis yielding four different population sets (PS1: 4A; PS2: 4B; PS3:45C; PS4: 4D).
(TIFF)

S5 Fig Manhattan plots of the physical position of SNPs (*x-*axis) and their correspondent *q*-values (log-transformed at *y*-axis) for inferring positive selection in Chromosome 6. The sliding-window *q*-value is indicated by a yellow continuous line. With a False Discovery Rate of 0.1 (dashed line), SNPs are color-coded according to the best supported model of selection, namely neutrality (black), selection in Africa (blue), selection in the Americas (green), and in both continents (convergent evolution, red). When an outlier SNP was located less than 50 kb apart from a gene, the closest gene name was written next to it. Different sets of populations were used in the analysis yielding four different population sets (PS1: 5A; PS2: 5B; PS3: 5C; PS4: 5D).
(TIFF)

S6 Fig Manhattan plots of the physical position of SNPs (*x-*axis) and their correspondent *q*-values (log-transformed at *y*-axis) for inferring positive selection in Chromosome 7. The sliding-window *q*-value is indicated by a yellow continuous line. With a False Discovery Rate of 0.1 (dashed line), SNPs are color-coded according to the best supported model of selection, namely neutrality (black), selection in Africa (blue), selection in the Americas (green), and in both continents (convergent evolution, red). When an outlier SNP was located less than 50 kb apart from a gene, the closest gene name was written next to it. Different sets of populations were used in the analysis yielding four different population sets (PS1: 6A; PS2: 6B; PS3: 6C; PS4: 6D).
(TIFF)

S7 Fig Gene sets significantly enriched for signals of selection. Nodes are gene sets that score a q-value ≤ 20% (before pruning). Gene sets are connected when at least one of them shares >33% of its genes with the other set. The width of the connecting lines represents the amount of similarity between sets; the size of a node scales with the size of the gene set. Each quadrant of a node represents results on one of the four selection scores, while the four rings in a node correspond to the four population sets (different combination of populations; see Methods). The color of each quarter of a ring corresponds to the significance of the test result (dark colored: q≤10%, light colored: 10%<q≤20%, white: q>20%). Note that gene sets that score high in the test using the probability for 'any selection' are not automatically scoring significant in the separate selection models and vice versa. A gene can have a high probability for 'any selection' but this can be the sum of relatively low probabilities of the three selection models 'selection in Africa', 'selection in America' and 'convergent selection'.
(EPS)

S1 File List with the outlier SNPs after sliding-windows. Information on SNP coordinates, α values for each model of selection (Africa, the Americas, or convergence), *q*-value, and nearest gene (NA stand for those cases where no gene was found in < 50 kb) is organized by sheet for each population set (PS).
(XLSX)

S1 Table Geographical origin of the populations considered in this study. (DOCX)

S2 Table Outlier non-synonymous SNPs showing significant directional positive selection signals in African and Amerindian populations suggesting adaptation to tropical forests. (DOCX)
