# Supplementary figures and images for: Detection of Convergent Genome-Wide Signals of Adaptation to Tropical Forests in Humans
Source: PLoS One. 2015 Apr 7;10(4):e0121557. doi: 10.1371/journal.pone.0121557 (PMC4388690; doi:10.1371/journal.pone.0121557)

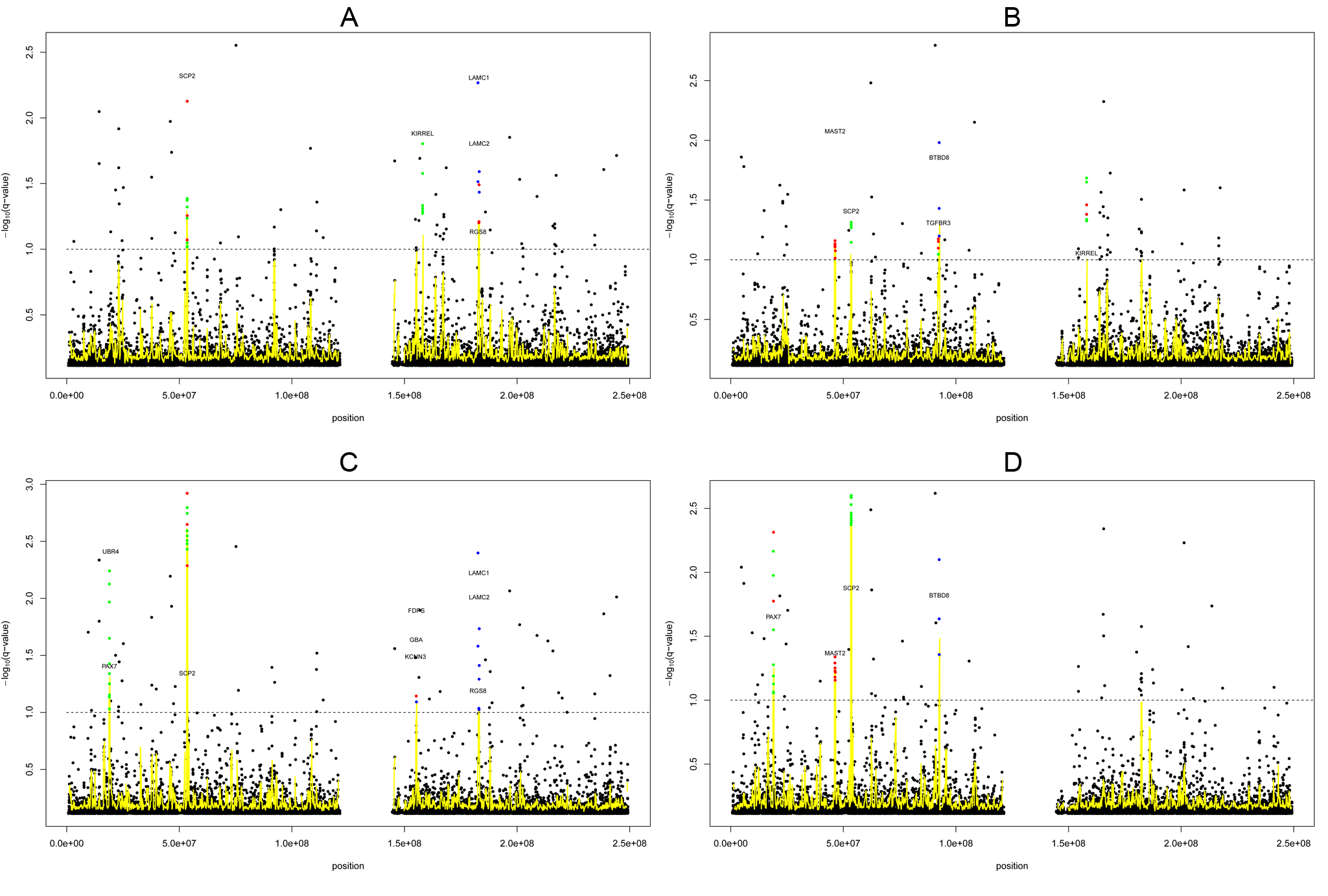

Supplement: S1 Fig — The sliding-window q-value is indicated by a yellow continuous line. With a False Discovery Rate of 0.1 (dashed line), SNPs are color-coded according to the best supported model of selection, namely neutrality (black), selection in Africa (blue), selection in the Americas (green), and in both continents (convergent evolution, red). When an outlier SNP was located less than 50 kb apart from a gene, the closest gene name was written next to it. Different sets of populations were used in the analysis yielding four different population sets (PS1: 1A; PS2: 1B; PS3: 1C; PS4: 1D). (TIFF) [file pone.0121557.s001.tiff]

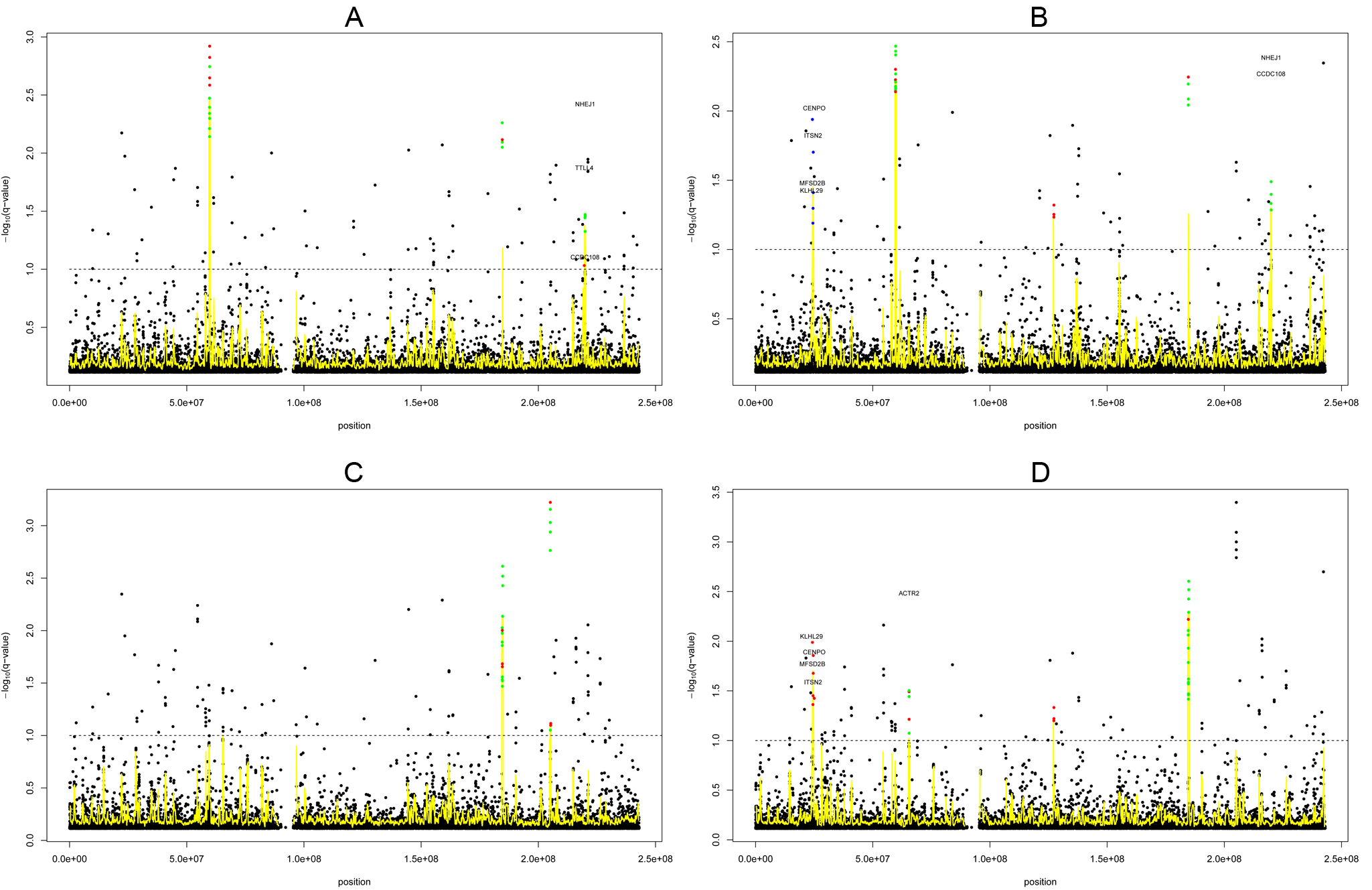

Supplement: S2 Fig — The sliding-window q-value is indicated by a yellow continuous line. With a False Discovery Rate of 0.1 (dashed line), SNPs are color-coded according to the best supported model of selection, namely neutrality (black), selection in Africa (blue), selection in the Americas (green), and in both continents (convergent evolution, red). When an outlier SNP was located less than 50 kb apart from a gene, the closest gene name was written next to it. Different sets of populations were used in the analysis yielding four different population sets (PS1: 2A; PS2: 2B; PS3: 2C; PS4: 2D). (TIFF) [file pone.0121557.s002.tiff]

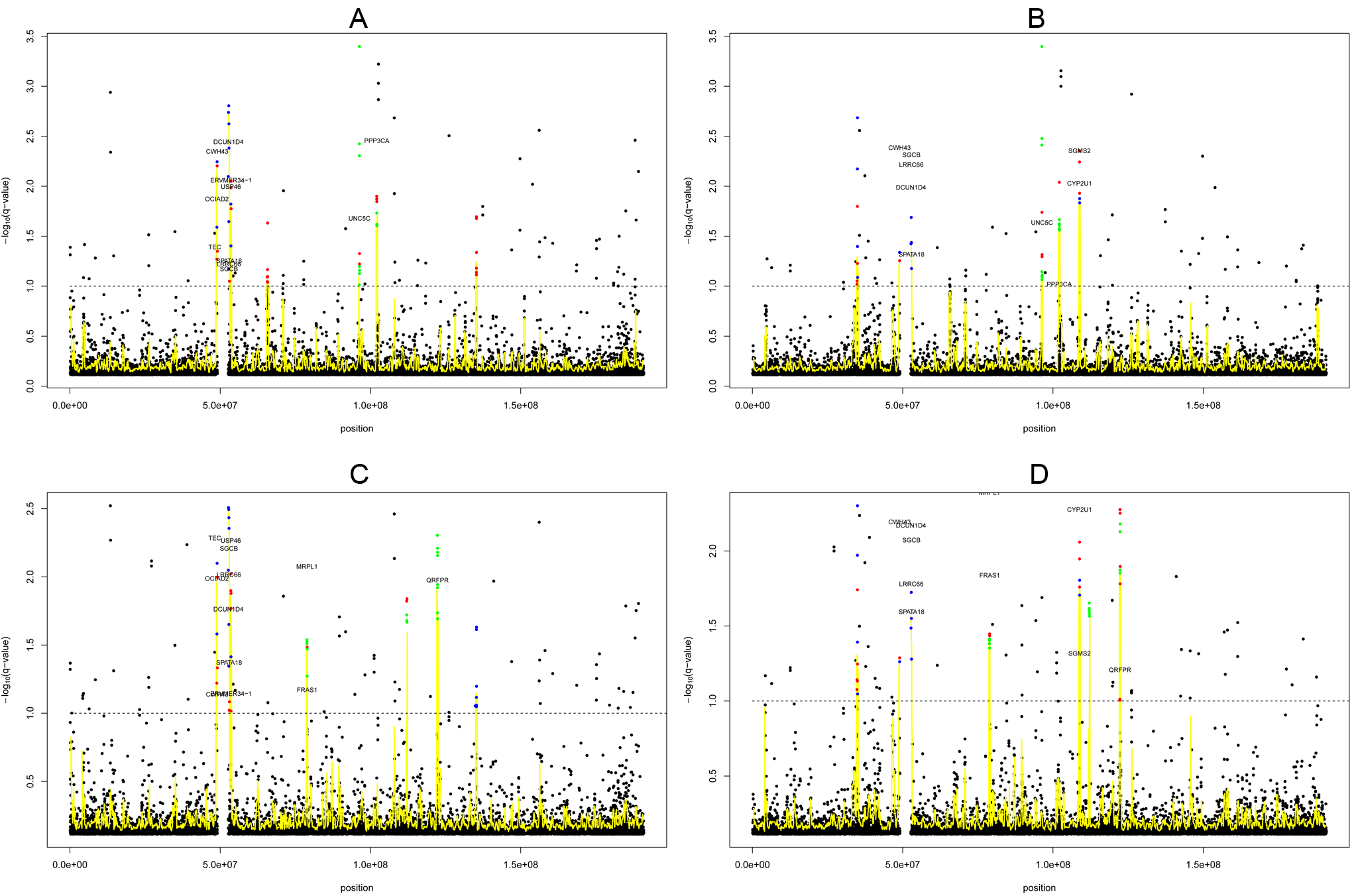

Supplement: S3 Fig — The sliding-window q-value is indicated by a yellow continuous line. With a False Discovery Rate of 0.1 (dashed line), SNPs are color-coded according to the best supported model of selection, namely neutrality (black), selection in Africa (blue), selection in the Americas (green), and in both continents (convergent evolution, red). When an outlier SNP was located less than 50 kb apart from a gene, the closest gene name was written next to it. Different sets of populations were used in the analysis yielding four different population sets (PS1: 3A; PS2: 3B; PS3: 3C; PS4: 3D). (TIFF) [file pone.0121557.s003.tiff]

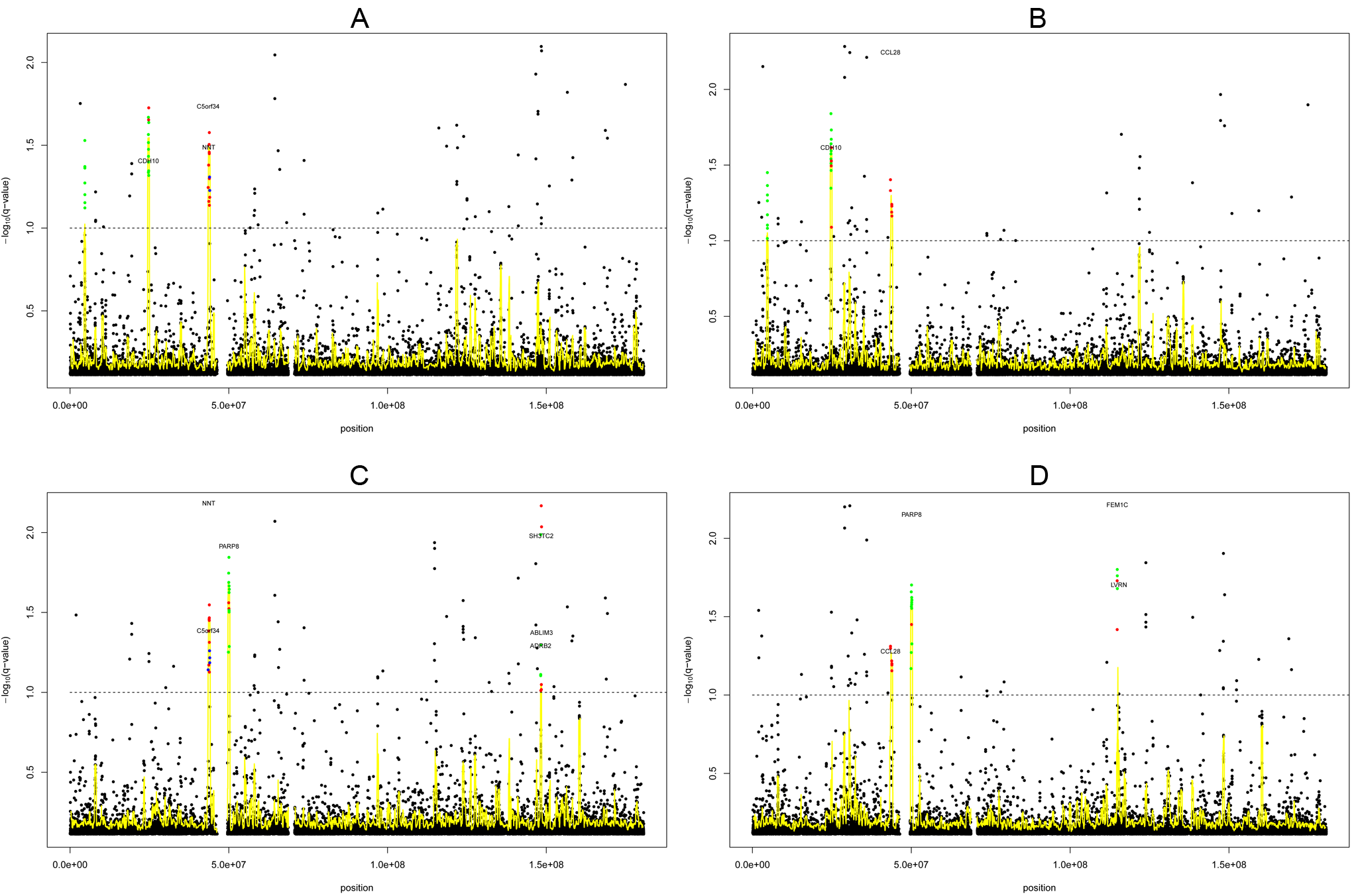

Supplement: S4 Fig — The sliding-window q-value is indicated by a yellow continuous line. With a False Discovery Rate of 0.1 (dashed line), SNPs are color-coded according to the best supported model of selection, namely neutrality (black), selection in Africa (blue), selection in the Americas (green), and in both continents (convergent evolution, red). When an outlier SNP was located less than 50 kb apart from a gene, the closest gene name was written next to it. Different sets of populations were used in the analysis yielding four different population sets (PS1: 4A; PS2: 4B; PS3:45C; PS4: 4D). (TIFF) [file pone.0121557.s004.tiff]

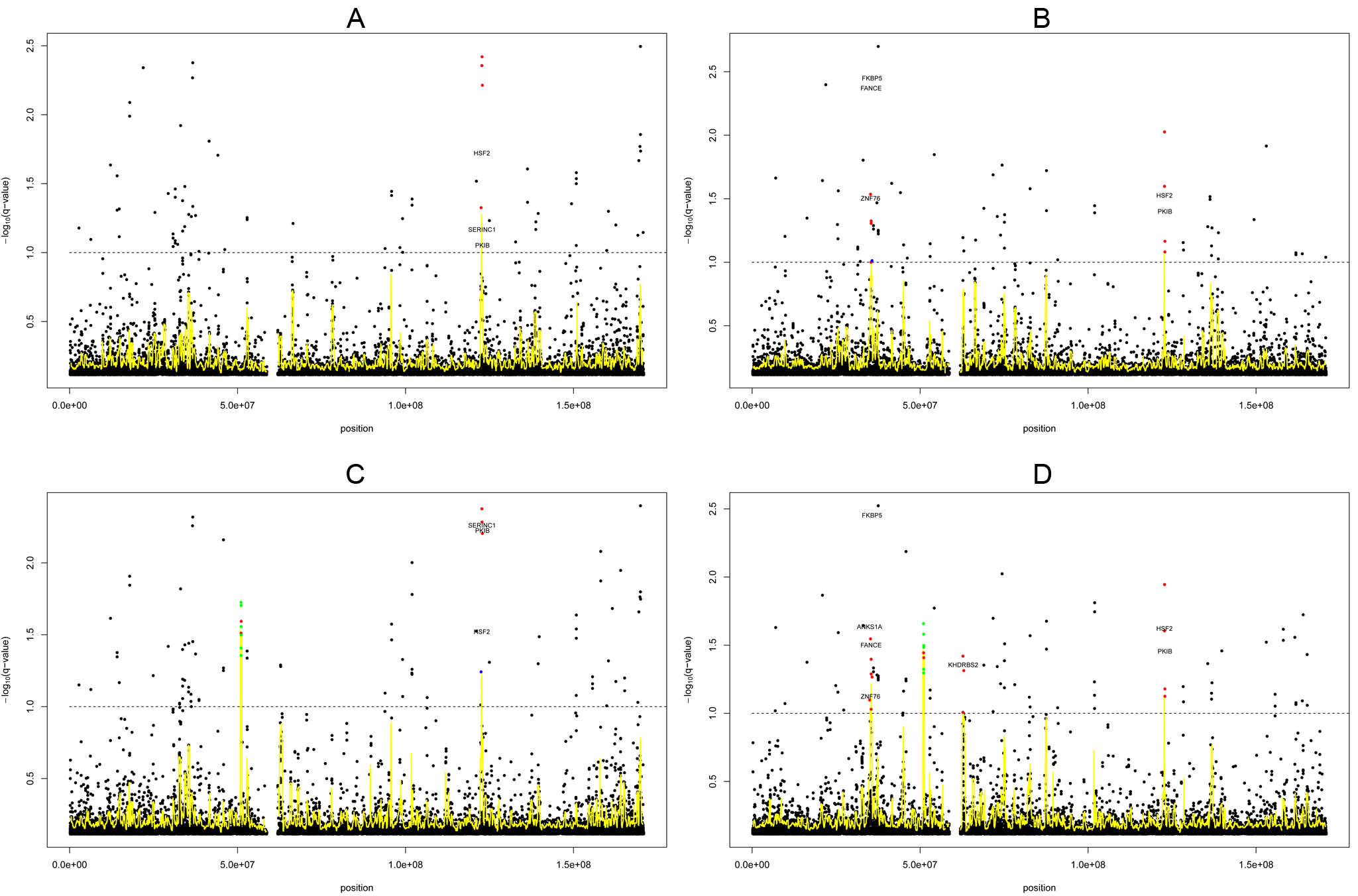

Supplement: S5 Fig — The sliding-window q-value is indicated by a yellow continuous line. With a False Discovery Rate of 0.1 (dashed line), SNPs are color-coded according to the best supported model of selection, namely neutrality (black), selection in Africa (blue), selection in the Americas (green), and in both continents (convergent evolution, red). When an outlier SNP was located less than 50 kb apart from a gene, the closest gene name was written next to it. Different sets of populations were used in the analysis yielding four different population sets (PS1: 5A; PS2: 5B; PS3: 5C; PS4: 5D). (TIFF) [file pone.0121557.s005.tiff]

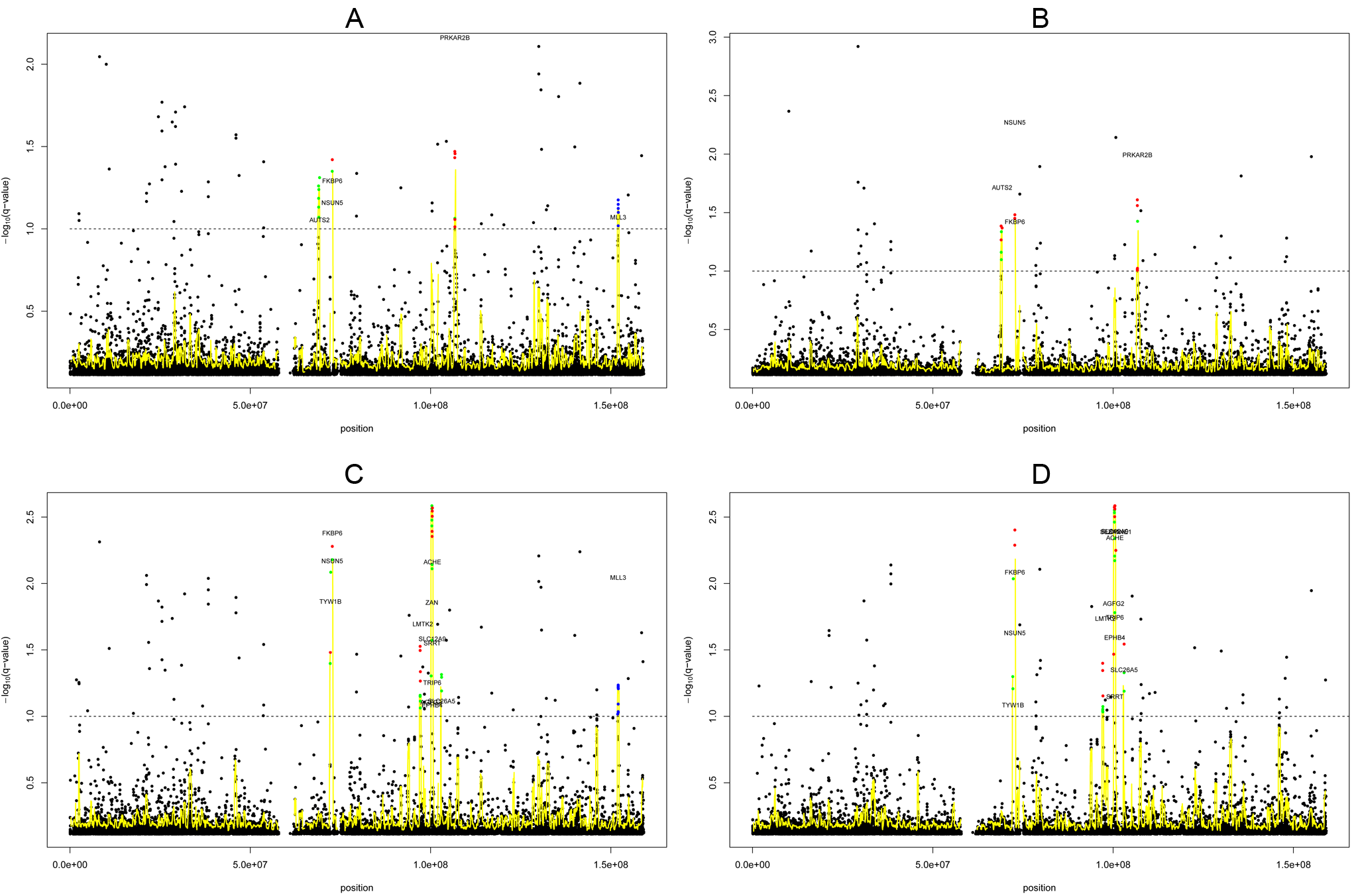

Supplement: S6 Fig — The sliding-window q-value is indicated by a yellow continuous line. With a False Discovery Rate of 0.1 (dashed line), SNPs are color-coded according to the best supported model of selection, namely neutrality (black), selection in Africa (blue), selection in the Americas (green), and in both continents (convergent evolution, red). When an outlier SNP was located less than 50 kb apart from a gene, the closest gene name was written next to it. Different sets of populations were used in the analysis yielding four different population sets (PS1: 6A; PS2: 6B; PS3: 6C; PS4: 6D). (TIFF) [file pone.0121557.s006.tiff]
