# Supplementary material for: Detection of Convergent Genome-Wide Signals of Adaptation to Tropical Forests in Humans
Source: PLoS One. 2015 Apr 7;10(4):e0121557. doi: 10.1371/journal.pone.0121557 (PMC4388690; doi:10.1371/journal.pone.0121557)
Supplement: S1 Table — (DOCX) [file pone.0121557.s009.docx]

**Table S1. Geographical origin of the populations considered in this study^a^.**

| **Population** | **Geographic origin** | **Coordinates of sample** |
| --- | --- | --- |
| Biaka Pygmy | Central African Republic | 4N, 17E |
| Karitiana | Brazil | 10S, 63W |
| Mandenka | Senegal | 12N, 12W |
| Mbuti Pygmy | Democratic Republic of Congo | 1N, 29E |
| Pima | Mexico | 29N, 108W |
| Surui | Brazil | 11S, 62W |
| Yoruba | Nigeria | 6-10N, 2-8E |

^a^Source: Cann et al. [10].
