# Supplementary material for: Detection of Convergent Genome-Wide Signals of Adaptation to Tropical Forests in Humans
Source: PLoS One. 2015 Apr 7;10(4):e0121557. doi: 10.1371/journal.pone.0121557 (PMC4388690; doi:10.1371/journal.pone.0121557)
Supplement: S2 Table — (DOCX) [file pone.0121557.s010.docx]

**Table S2. Outlier non-synonymous SNPs showing significant directional positive selection signals in African and Amerindian populations suggesting adaptation to tropical forests.**

| **SNP** | **Chromosome** | **Selection^a^** | **Gene** |
| --- | --- | --- | --- |
| rs11211247 | 1 | Convergence (PS2, PS4) | *MAST2* |
| rs20563 | 1 | Convergence (PS1), Africa (PS3) | *LAMC1* |
| rs2114664 | 2 | Convergence (PS1) | *TTLL4* |
| rs6736922 | 2 | Americas (PS1, PS2) | *CCDC108* |
| rs7603997 | 2 | Africa (PS2), Convergence (PS4) | *ITSN2* |
| rs2302310 | 4 | Convergence (PS4) | *QRFRP* |
| rs6875902 | 5 | Convergence (PS3) | *SH3TC2* |
| rs2075756 | 7 | Convergence (PS3, PS4) | *TRIP6* |
| rs10953303 | 7 | Americas (PS3, PS4) | *ZAN* |
| rs2293767 | 7 | Americas (PS3, PS4) | *ZAN* |
| rs6942733 | 7 | Americas (PS3, PS4) | *ZAN* |
| rs2271904 | 10 | Africa (PS2, PS4) | *ECD* |
| rs3812619 | 10 | Africa (PS2, PS4) | *ECD* |
| rs4294502 | 10 | Africa (PS2, PS4) | *TTC18* |
| rs667782 | 11 | Americas (PS4) | *PUS3* |
| rs10507047 | 12 | Americas (PS3, PS4) | *FGD6* |
| rs7135194 | 12 | Convergence (PS3), Americas (PS4) | *FGD6* |
| rs2075090 | 19 | Americas (PS3, PS4) | *ZNF862* |
| rs5997917 | 22 | Convergence (PS3), Americas (PS4) | *LIMK2* |

^a^Directional positive selection in Africa, the Americas or both continents (convergence) for the corresponding population set (PS) in parenthesis.
